# The influence of digital professional development and professional learning communities in the relationship between school digital preparedness and digital instructional integration

**DOI:** 10.1371/journal.pone.0328883

**Published:** 2025-07-28

**Authors:** Stephen Kwashie Amemasor, Stephen Opoku Oppong, Benjamin Ghansah, Ben-Bright Benuwa, Mathias Agbeko

**Affiliations:** Department of ICT Education, University of Education, Winneba, Ghana; King Saud bin Abdulaziz University for Health Sciences, SAUDI ARABIA

## Abstract

Integration of digital tools and resources in STEM instruction has garnered significant attention due to its high potential. Digital professional development and professional learning communities are identified as a pivotal factor for equipping teachers with the necessary digital skills to effectively orchestrate digital resources. Notably, their role is considered critical. However, the intricate relationships among school digital preparedness, professional learning community, digital professional development, and digital instructional integration among STEM teachers remain underexplored. Utilising partial least-squares–structural equation models (PLS–SEM), the present study examined links in school digital preparedness, professional learning community, digital professional development, and digital instructional integration among STEM teachers (N = 188). Findings from the PLS–SEM analysis indicate that digital professional development and professional learning community exhibit a direct positive relationship with school digital preparedness and digital instructional integration. Relatedly, digital professional development is positively correlated with digital instructional integration. In terms of indirect effect, findings show that professional learning community and digital professional development play a significant positive mediating role in linking digital professional development and digital instructional integration. This study reports new evidence on the influence of school digital preparedness on digital instructional integration through digital professional development among 188 STEM teachers and concludes that, when STEM teachers regularly immerse themselves in digital professional development program, they are more likely to benefit from their digital professional development by integrating digital technologies in classroom instruction. Policymakers and educational leaders should consider promoting digital professional development among STEM teachers, along with efforts to encourage digital instructional integration.

## Introduction

Efforts to adapt education to the evolving demands of modern society increasingly focus on integrating digital technology into classrooms across all levels and disciplines [1–3]. Digital integration involves embedding technology seamlessly into teaching and learning processes, transforming traditional methods into interactive and dynamic experiences. This goes beyond basic uses, such as presentations or online research, to strategically apply technology to achieve specific educational objectives. By leveraging digital tools, educators can foster collaboration, engage students actively, and create tailored learning experiences that improve understanding. The primary aim of digital integration is to equip students with the skills they need to thrive in a world where technology profoundly influences nearly every aspect of life.

Incorporating digital tools is particularly critical in STEM (Science, Technology, Engineering, and Mathematics) education. Research indicates that when STEM teachers use digital games, interactive simulations, and advanced modelling tools, students gain a deeper comprehension of complex concepts [4]. This approach enhances knowledge acquisition and nurtures essential skills like data analysis, computational thinking, and problem-solving [5]. Furthermore, digital integration mirrors the interdisciplinary and collaborative nature of STEM professions, preparing students for modern workplaces where teamwork is key [6]. Studies also show that students perform better academically when they use digital tools, such as iPads, in learning environments designed to make lessons more engaging and relevant [7].

Countries like Ghana have been working to align their education systems with global technological advancements, recognising the need to equip teachers with the necessary digital competencies [8,9]. Teachers play an indispensable role in education, as they serve as the primary implementers of policies in the classroom. The research underscores that the presence of highly skilled teachers can significantly boost student performance, especially for marginalized students who rely on schools for access to digital resources [10,11]. Providing teachers with digital skills not only improves STEM education but also narrows the achievement gap for students with limited access to technology outside school.

The success of digital integration in education hinges on the preparedness of schools. School digital preparation involves providing robust infrastructure, including high-speed internet, up-to-date hardware, and reliable software, to create an environment conducive to digital learning [12,13]. Additionally, adequate technical support and maintenance are vital to minimizing disruptions and enabling teachers to focus on instructional delivery [14]. Schools must also ensure that their curricula align with technological advancements, integrating digital tools to enhance engagement and retention among students. Addressing issues of equity and access is equally critical, as it ensures that all students, regardless of socioeconomic background, can benefit from digital learning tools.

Leadership within schools plays a pivotal role in digital preparedness. School leaders are tasked with establishing a clear vision for technology use, allocating resources strategically, and fostering a culture of innovation and continuous improvement [12]. Effective school administrators prioritize building strong digital infrastructure, providing ongoing professional development for teachers, and ensuring equitable access to resources for all students. By addressing these areas, school leaders create inclusive and technologically advanced environments that support high-quality education [15].

Teacher training is another critical component of digital integration. Professional development programs focused on digital competencies empower teachers to design interactive and engaging lessons that cater to diverse student needs [16]. Teachers who participate in these programs gain the skills necessary to adapt to rapidly changing technologies and teaching practices [17]. Research shows that schools with consistent digital training initiatives see greater teacher readiness for digital integration compared to those without such opportunities [18,19]. These training activities often take flexible forms, including online workshops, virtual meetings, and in-person conferences, making it easier for teachers to participate despite busy schedules [20]. By fostering a network of educators who share strategies and address challenges collaboratively, professional development programs enhance teachers’ ability to create inclusive and effective learning environments.

Professional Learning Communities (PLCs) provide an additional platform for supporting digital integration. These collaborative frameworks bring together teachers and school leaders to improve teaching practices and student outcomes [21]. Within PLCs, educators share ideas, discuss teaching challenges, and develop solutions collectively. This culture of collaboration and shared responsibility enhances instructional quality and fosters continuous improvement [22]. PLCs are particularly effective for digital integration, enabling teachers to learn from each other’s experiences and adapt innovative practices more quickly [23]. With the growth of online platforms, PLCs have expanded to virtual spaces, allowing educators from diverse locations to connect, share insights, and solve problems collaboratively [24]. These online communities remove barriers such as time constraints and transportation issues, making professional collaboration more accessible and inclusive.

The integration of digital technology into education brings numerous benefits. It enhances learning by making lessons more engaging and interactive, especially in complex subjects like STEM. It equips students with essential 21st-century skills, including problem-solving and teamwork, while also addressing disparities in access to resources. Teachers, empowered through professional development and collaborative communities, gain confidence and expertise in using technology effectively. However, challenges such as infrastructure gaps, lack of teacher training, and inequitable access must be addressed to realize the full potential of digital integration.

By investing in robust digital infrastructure, supporting teachers through continuous professional development, and fostering collaborative environments like PLCs, schools can create dynamic and future-ready learning spaces. As technology continues to shape the world, such efforts ensure that students are well-prepared to succeed in a rapidly evolving digital landscape

To delve into digital integration issues, the research is guided by the following research questions:

RQ1: What is the current level of digital preparedness among senior high schools in the three Tongu districts and how does it affect digital integration among STEM teachers?

RQ2: How does digital professional development influence the integration of digital technologies in STEM instruction among senior high school teachers in the three Tongu districts?

RQ3: What role do professional learning communities play in supporting digital instructional integration?

RQ4: How do digital professional development and professional learning communities interact to influence the relationship between school digital preparedness and digital instructional integration?

## Literature review

Adapting education to meet the changing demands of modern society has increasingly focused on integrating digital technology into classrooms across all levels of learning. Digital integration refers to embedding technology seamlessly into teaching and learning processes to [transform traditional methods into interactive, dynamic experiences [25,26]. This approach extends beyond the basic use of technology for tasks such as presentations or online research, emphasizing strategic application to achieve specific educational objectives. By leveraging digital tools, educators can promote collaboration, actively engage students, and create personalized learning experiences that enhance comprehension [27]. The primary goal of digital integration is to equip students with the technological competencies necessary for success in an increasingly digital world.

### STEM education

The term STEM, introduced by the National Science Foundation (NSF), stands for Science, Technology, Engineering, and Mathematics. It groups these disciplines to represent fields focused on innovation and problem-solving, though some debate exists about treating them as a unified area. STEM education has gained significant attention because it prepares students for the modern workforce by developing critical thinking, problem-solving, and teamwork skills. At its core, STEM education is interdisciplinary, combining knowledge and skills from each subject into integrated learning experiences. It emphasizes real-world applications, helping students connect theoretical concepts with practical use. For example, Tytler emphasized the importance of applying STEM knowledge to real-life challenges [28], and Widya et al. advocated for reducing boundaries between the disciplines to foster a more holistic understanding [29].

Research shows that STEM education positively impacts students by improving problem-solving abilities, cognitive skills, and adaptability to technological changes. It also equips them with the creativity and analytical skills needed for STEM-related careers, which are critical for innovation, economic growth, and global competitiveness [30,31]. To maximize STEM education’s benefits, experts recommend integrating digital tools to engage students more effectively. These tools encourage collaborative problem-solving, fostering creativity and analytical thinking. However, challenges like teacher preparedness and ensuring equitable access must be addressed to make STEM education accessible to all. Continuous improvement in teaching methods is essential to ensure every student benefits from quality STEM learning opportunities.

### Digital instructional integration

The rapid development of digital technologies has significantly transformed teaching and learning by offering innovative tools that improve educational practices. These technologies, including online interactive learning platforms, virtual reality (VR), simulations, and digital gaming, have a notable impact, particularly in STEM education. Many countries are prioritizing the integration of digital tools in both teaching and assessment because today’s students are deeply immersed in the digital world, regularly using technology on their own. Research suggests that incorporating digital technology into education fosters collaboration between students and teachers, making it easier to achieve learning goals [32].

Digital tools like interactive whiteboards, learning management systems (LMS), and digital textbooks have revolutionized traditional classrooms, creating more dynamic and engaging learning environments. These tools allow for a deeper understanding of subjects through multimedia resources and interactive activities. Emerging technologies like augmented reality (AR) and virtual reality (VR) provide immersive experiences that are especially beneficial for subjects requiring spatial understanding, such as science and engineering [33]. These technologies allow students to visualize complex concepts and conduct virtual experiments, enhancing engagement and comprehension. Artificial intelligence (AI) is also making a significant impact on education by offering personalized learning experiences. AI systems analyze student data to customize lessons and provide instant feedback, addressing individual learning needs [34]. AI can also identify learning gaps and suggest resources, helping students progress at their own pace and ensuring they receive the support needed to achieve their goals [35,36].

However, despite these benefits, the integration of digital technologies in education also presents challenges. A major issue is the “digital divide,” which refers to the gap between those who have access to technology and those who do not. This divide can worsen educational inequalities, as students without reliable internet or modern devices face significant disadvantages [37]. Addressing this gap requires cooperation among lawmakers, educators, and technology providers to ensure equal access to digital resources for all students [38].

Another challenge is the need for teachers to develop digital skills to effectively incorporate technology into their teaching methods [39,40]. In conclusion, while digital educational technologies offer substantial benefits such as increased interactivity, personalized learning, and greater accessibility, it is essential to address the digital divide and ensure that teachers are adequately trained to make full use of these new technologies.

### School digital preparedness and digital instructional integration

Prior studies emphasize the substantial impact of a school’s digital preparedness on the incorporation of digital technology into classroom instruction and learning procedures [41,42]. School digital preparation refers to the policies implemented by school administrators to promote the successful integration of digital technology, as well as the availability of digital equipment and facilities that enhance instructional activities [43]. Previous research has emphasized that being well-prepared in digital technology is crucial for influencing teachers’ attitudes and abilities in integrating technology into their teaching [44]. Factors such as digital culture, infrastructure, and resources play a significant role in determining the success of digital integration. Additional research confirms a direct correlation between the technical assistance and services provided by a school and teachers’ endeavours to incorporate digital technology [45]. These findings indicate that creating an environment in schools that fosters innovation can greatly improve the incorporation of digital technologies, leading to better quality of teaching and learning in a world that is becoming increasingly digitalized [46]. Furthermore, research indicates that the incorporation of digital technology should support and improve current teaching methods; therefore, there should be the provision of readily available technical support that would help teachers in terms of technological glitches [47]. Because teachers are sometimes new to emerging educational technologies, providing them with a support system would assure them that, when they face difficulties, they could get assistance, and this would ensure robust digital instructional integration given the rapid evolution of digital technologies. As a result, there is a cultivation of a culture of innovation and ongoing enhancement in educational institutions to ensure that digital integration initiatives stay pertinent and efficiently cater to the varied requirements of learners [48].

### School digital preparedness and digital professional development

The connection between a school’s digital preparedness and its approach to digital professional development is becoming increasingly important for ensuring effective digital integration in education. Digital preparedness goes beyond simply having the right technology; it involves creating an environment that supports continuous learning and adaptation. This means that schools must not only provide teachers with the necessary tools and software but also establish a culture that encourages ongoing professional growth [49]. Digital professional development is key to this process, as it equips teachers with the skills and knowledge to use digital tools effectively in their teaching [50].

Studies show that schools that prioritize digital preparedness are more likely to implement comprehensive professional development programs aimed at improving teachers’ digital competencies. These programs typically include training on new technologies, digital teaching methods, and how to incorporate these tools into the curriculum [51]. Research by Örnek et al. indicates that schools with strong digital strategies often offer workshops, mentoring, and collaborative projects to help teachers explore and use digital tools [52]. This kind of environment fosters a culture of innovation and continuous learning, which is critical for successful digital integration in education.

Pérez-Jorge et al. emphasize that schools can enhance teachers’ engagement in professional development by investing in digital infrastructure and providing technical support [53]. This enables teachers to apply what they learn and use digital tools to improve teaching and learning outcomes. When schools align their digital readiness with professional development strategies, they ensure that teachers not only learn about new technologies but also become proficient in using them effectively in the classroom [54]. This alignment helps bridge the gap between the availability of technology and its effective use, ensuring that teachers are confident and well-supported as they integrate digital tools into their teaching practices.

Gonzalez et al. highlight the importance of customizing digital professional development programs to meet the specific needs of teachers and the unique context of each school [55]. Tailoring training to teachers’ daily practices ensures that it is relevant and immediately applicable. Schools with the necessary digital infrastructure are better equipped to understand these needs and design focused training programs that not only improve teachers’ proficiency but also encourage them to engage in continuous learning and problem-solving [56].

In conclusion, the relationship between school digital preparedness and digital professional development is mutually reinforcing. Schools that are digitally prepared are better able to support professional development initiatives, which, in turn, help teachers effectively use digital tools in their instruction. This dynamic is essential for achieving successful digital integration and improving educational outcomes

### School digital preparedness and professional learning communities

The relationship between a school’s digital preparedness and the presence of professional learning communities (PLCs) is an important factor in the successful integration of digital technology in education. Digital preparedness refers to the infrastructure, policies, and resources a school has to support digital learning, such as reliable internet, digital devices, and the ability to support collaborative teaching practices [57]. PLCs, on the other hand, are groups of teachers who work together to improve their teaching methods and student outcomes through shared learning and reflection [58]. When schools are digitally prepared, they provide a strong foundation for PLCs to thrive, allowing teachers to effectively use digital tools in their teaching.

Research shows that schools with robust digital infrastructure are better equipped to support PLCs focused on integrating technology. These schools provide the resources needed for teachers to collaborate, share digital tools, and continually improve their understanding of technology in education [59]. Viberg et al. highlight that digital preparedness includes providing essential resources like internet access, digital devices, and collaborative platforms, which enable teachers to work together and enhance their digital teaching practices [60]. Additionally, schools that prioritize digital readiness foster a culture of creativity and collaboration among teachers. A supportive digital environment helps teachers openly share their experiences, challenges, and successes in using technology [61]. This collaboration not only improves teaching methods but also strengthens the sense of community and shared goals among teachers. Furthermore, when teachers collaborate in a digitally-equipped environment, they can co-create and refine digital teaching strategies that benefit students’ academic performance [62].

Moreover, digitally prepared schools often offer professional development opportunities aligned with the goals of PLCs. This ensures that teachers gain both the knowledge of digital tools and the confidence to use them collaboratively to improve their teaching methods [63]. Digital tools also enhance the effectiveness of PLCs by enabling communication and collaboration among teachers, even across different locations [64]. This is particularly beneficial in larger or geographically dispersed schools, where digital platforms can compensate for limited in-person interactions.

In conclusion, the synergy between a school’s digital preparedness and its PLCs is essential for successful digital integration. Digitally-equipped schools provide the infrastructure and resources that enable PLCs to thrive, while PLCs use these tools to enhance teaching and improve student outcomes. The combined efforts of digital preparedness and collaborative learning create a culture of continuous improvement and innovation, which is critical for integrating digital technology into education.

### Digital professional development and digital instructional integration

Research has shown that there is a strong connection between how often teachers participate in professional development activities and how effectively they use digital tools in their teaching. Professional development programs help teachers improve their teaching methods, and when focused on digital tools, these programs can greatly enhance how technology is integrated into classrooms, especially for STEM (Science, Technology, Engineering, and Math) education. This is because STEM subjects require the use of technology, and digital professional development equips teachers with the skills needed to integrate these tools effectively [65,66]. Studies highlight that digital professional development is a key factor in helping teachers integrate digital tools into their lessons. Teachers who take part in thorough training programs are more likely to gain the skills and confidence necessary to use digital tools in their teaching. These programs help teachers develop digital competencies, which include the ability to choose the right digital tools for their lessons and understand how different tools can work together to enhance teaching [67,68].

When teachers attend training that exposes them to new digital technologies, they are more likely to use these technologies in their classrooms. This makes their teaching more in line with modern educational trends, which increasingly involve the use of digital tools. Research also suggests that the more hours teachers spend in training on digital tools, the more likely they are to use them in their teaching [69]. Therefore, it’s not enough to have a one-time training session; digital professional development needs to be continuous and timely, ensuring that teachers stay up to date with new technologies and teaching methods [70].

Digital professional development programs often focus on both technical skills and pedagogical practices. Teachers learn not only how to use digital tools but also how to apply them in ways that improve student learning. This kind of development makes it easier for teachers to shift from traditional teaching methods to digital ones, providing them with the skills and knowledge they need to incorporate technology into their lessons effectively [71,72]. In some schools, digital professional development is also referred to as “digital instructional coaching.” This approach helps teachers build their teaching competencies and enhances learning in STEM subjects [73]. Continuous professional development ensures that teachers remain informed about the latest technological advances and teaching techniques. Since digital tools and teaching methods are constantly evolving, teachers must keep improving their skills to stay relevant and effective [74,75].

Overall, digital professional development is vital in helping teachers integrate digital technologies into their teaching. By continually enhancing their skills, teachers can adapt to changes in technology and incorporate new tools into their lessons, which ultimately benefits students’ learning outcomes. In summary, regular, ongoing professional development is essential for teachers to confidently and competently use digital tools, ensuring that their teaching methods align with the needs of modern education.

### Professional learning communities and digital instructional integration

Professional learning communities (PLCs) play a significant role in enhancing teaching by fostering collaboration among teachers. These communities help teachers build a shared vision and focus on creative teaching methods, which helps improve their teaching practices [76]. A PLC is a group where teachers can work together to improve their teaching and share experiences and ideas about using digital tools in the classroom [77]. This collaborative approach helps create a culture of continuous learning and innovation, encouraging teachers to try new technologies and improve their methods.

In PLCs, teachers work together to solve common challenges they face in their teaching. They may meet in person or online to discuss ways to integrate digital tools into their lessons [78]. By sharing ideas and supporting each other, teachers can overcome obstacles and develop better strategies for using digital resources effectively in their classrooms. Research shows that when teachers engage in reflective and collaborative discussions within PLCs, they improve their teaching skills and knowledge [79].

The concept of capacity building is important within PLCs. It combines motivation, skills, a positive learning environment, and support to create effective teaching practices [80]. In recent years, PLCs have become more popular, sometimes referred to as “teacher networking,” where teachers share knowledge and teaching methods [81]. Studies have highlighted the positive impact of PLCs on teachers’ attitudes towards using digital tools in their teaching. PLCs help teachers address the challenges they face while integrating digital technologies into their classrooms [82,83]. Research also shows that when teachers work in communities that encourage idea-sharing and learning, they are more successful in integrating digital tools into their instruction [84]. Teachers are more likely to see digital tools as valuable if their colleagues also value them and share their experiences with them [85].

**Fig 1:**
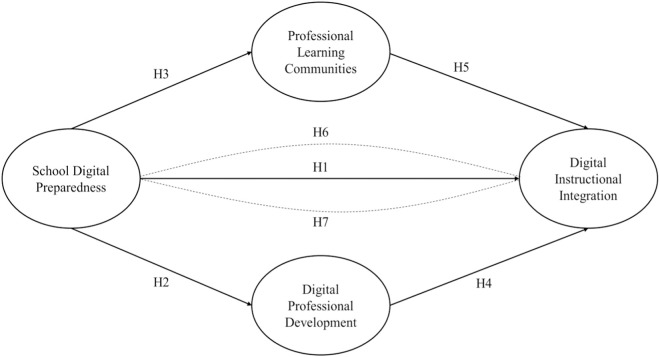
Hypothetical Framework of relationship among School Digital Preparedness, digital professional development, professional learning communities and digital instructional integration.

To achieve this, the current research sets the following seven hypotheses (H) to guide the study as presented in the conceptual model in Fig 1 below.

H1: School digital preparedness (SDP) can positively and significantly affect digital instructional integration (DII) directly.

H2: School digital preparedness can positively and significantly affect digital professional development (DPD) directly.

H3: School digital preparedness can positively and significantly affect professional learning communities (PLCs) directly.

H4: Digital professional development has a significant positive influence on digital instructional integration directly.

H5: Professional learning communities can positively and significantly influence digital instructional integration directly.

H6: School digital preparedness can positively and significantly affect digital instructional integration through professional learning communities.

H7: School digital preparedness can positively and significantly affect digital instructional integration through digital professional development.

## Theoretical framework

### Community of practice (CoP) theory

**Fig 2:**
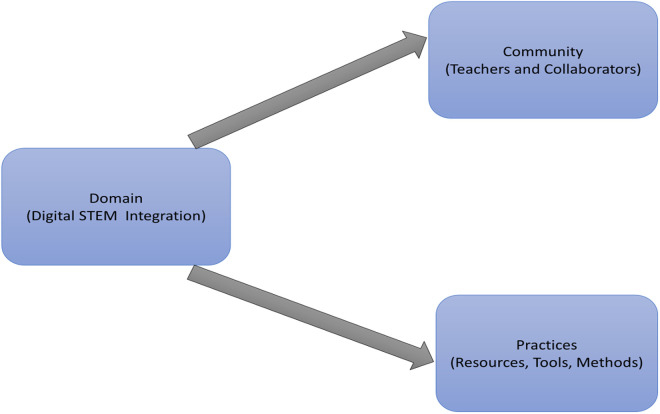
Community of Practice model.

The Community of Practice (CoP) theory, introduced by Wenger, emphasizes the importance of social learning that occurs through collaboration and shared practices among individuals with common interests and goals, as shown in Fig 2 [86]. According to CoP theory, learning is a social phenomenon that happens when people engage with each other in a community, exchanging knowledge and experiences to develop a collective understanding. The theory outlines two key processes: participation, which involves active engagement and interaction with peers, and reification, which refers to creating tangible outputs of knowledge, such as teaching materials or tools [87]. Through participation and reification, individuals not only learn new perspectives and skills but also contribute to the advancement of the community’s collective practices.

In educational settings, the CoP theory emphasizes the importance of teacher collaboration to enhance teaching methods. Teachers share resources, engage in professional discussions, and collaboratively solve problems, creating a supportive and nurturing environment. This environment encourages teachers to learn from each other, adapt to new challenges, and improve their effectiveness in the classroom [88]. Teachers working together in this manner foster continuous professional growth and enhance their ability to handle challenges, including integrating digital technologies into their teaching.

CoP theory has been applied in studies exploring how teachers collaborate in digital learning communities to improve their use of technology in classrooms [89,90]. Research shows that teachers in these collaborative settings are more likely to share ideas, enhance their digital skills, and explore new technologies together. This environment motivates teachers to reflect on their teaching methods, create innovative educational resources, and overcome obstacles related to technology integration. By solving problems together, teachers within a CoP can more effectively address challenges such as selecting suitable digital tools and aligning them with curriculum goals [91].

The CoP theory is especially relevant for examining the integration of digital technology in classrooms. It helps explain how professional learning communities and digital professional development contribute to improving teachers’ digital competencies [92–94]. In particular, CoP highlights the social dynamics of learning, where teachers interact with colleagues, share digital strategies, and collaboratively navigate the complexities of technology integration in education. The theory also emphasizes the role of these communities in fostering ongoing professional development, enabling teachers to adjust to the fast-changing digital landscape and ensuring they remain equipped to meet the demands of modern education.

This study utilizes CoP theory to explore how collaborative networks among senior high school STEM teachers in the three Tongu districts can enhance digital instructional integration. By examining how teachers in these communities work together, share digital tools, and address integration challenges, the study aims to investigate how CoP theory contributes to improving digital readiness in schools and ultimately enhancing STEM education. Through this framework, the study can uncover how CoP fosters a culture of continuous learning and adaptation, ensuring teachers are well-prepared to integrate technology into their teaching practices effectively.

## Research methodology

In this study, a quantitative research design will be adopted to explore the relationships between school digital preparedness, digital instructional integration, digital professional development (DPD), and professional learning communities (PLCs) among senior high school STEM teachers. The goal is to identify both direct and indirect effects, where digital professional development (DPD) may act as a mediator and PLCs as a moderator in the relationship between school digital preparedness and digital instructional integration. Given the intricate nature of these relationships and the need to analyse them in a way that allows for both flexibility and statistical robustness, Partial Least Squares Structural Equation Modelling (PLS-SEM) will be employed. PLS-SEM is a powerful statistical tool for analysing complex multivariate relationships, especially in educational and social science research where sample sizes are often limited, and data distributions may deviate from normality. PLS-SEM is also well-suited for predictive modelling, making it ideal for exploring how various factors influence digital instructional integration in this context.

The study involves multiple latent constructs, including school digital preparedness, DPD, PLCs, and digital instructional integration, as well as hypothesized mediation and moderation effects. PLS-SEM is adept at handling models with multiple paths and latent variables, making it well-suited for this study’s design. Unlike covariance-based SEM (CB-SEM), which generally requires larger samples, PLS-SEM can reliably estimate parameters with smaller sample sizes, making it suitable for studies where sample sizes may be constrained due to logistical or budgetary limitations. PLS-SEM is robust to data that does not meet normality assumptions. In educational research, survey responses often display skewed distributions; PLS-SEM accommodates this by using non-parametric methods, specifically bootstrapping, to assess the statistical significance of paths. PLS-SEM is primarily used for maximizing the explained variance in dependent variables (in this case, digital instructional integration) rather than just model fit, which aligns well with the research objective of predicting digital integration outcomes based on school preparedness, DPD, and PLCs.

In this research study, descriptive statistics will play a crucial role in providing a foundational understanding of the sample population and the primary variables: School Digital Preparedness, Digital Professional Development (PD), Professional Learning Communities (PLCs), and Digital Instructional Integration. These statistics help to assess the characteristics of the data, such as the central tendencies, variabilities, distributions, and response frequencies across various demographics and variables. The information provided through descriptive statistics will offer valuable insights and ensure the data are well-prepared for subsequent analyses in PLS-SEM. This quantitative research design with PLS-SEM will allow for a nuanced understanding of how school digital preparedness, DPD, and PLCs interact to influence digital instructional integration, informing strategies for enhancing digital practices in STEM education.

The study was approved by institutional review board and the need for consent was waived by the ethics committee.

### Instruments

The main instrument used to collect data for this study was a structured questionnaire that was delivered through the Kobo Collect Toolbox Survey. This was done from 1/07/2024–31/10/2024. Two separate surveys were created: one designed for teachers and another specifically tailored for headteachers. The headteachers’ questionnaire aimed to evaluate the level of preparedness of schools in terms of digital resources. It included questions that specifically addressed the availability and utilization of digital technologies, as well as the overall digital culture within the schools. The questions were modified from the PISA 2022 school questionnaire to ensure their relevance to the research population and environment. The questions addressed factors such as the ratio of students to digital devices, the presence of internet-connected laptops, interactive whiteboards, and data projectors. In addition, headteachers were requested to assess their level of agreement with statements concerning the school’s potential to improve teaching and learning using digital devices, encompassing the technical and pedagogical proficiency of teachers, access to digital resources, and the provision of technical assistance.

Relatedly, the data collection for teachers was conducted using a specifically prepared structured questionnaire that aimed to capture comprehensive data on all elements of their professional development. The purpose of this questionnaire was to collect information about the demographic characteristics of teachers, such as their age, gender, educational attainment, and length of teaching experience. The study also examined their professional development by investigating how often and in what manner they engaged in programs designed to improve their teaching abilities and incorporate digital technologies into their classrooms. In addition, the questionnaire assessed the level of collaboration among teachers by investigating the frequency of their sharing of teaching resources, discussing new educational technology, and collaborating to establish uniform criteria for student assessments.

To guarantee the questionnaire was relevant as well as effective for the targeted research population, the questions were modified from the PISA 2022 teacher questionnaire, which is a widely recognized international survey instrument. Nevertheless, the questions were modified to correspond with the particular circumstances of senior high schools in the North, South, and Central Tongu districts. This restructuring entailed altering the wording, examples, and response options to accurately represent the local educational setting and the distinct challenges and possibilities encountered by teachers in these areas. The survey consisted of a combination of closed-ended questions, which enabled respondents to provide specific and measurable answers, and Likert-scale items, which offered information about the frequency and intensity of different teaching approaches and attitudes towards digital integration. The utilization of various question types facilitated the acquisition of reliable, numerical data that could be methodically examined to detect recurring themes and tendencies in teachers’ methods of engaging in professional development, professional learning communities, and the integration of digital technology into their teaching practices. The population for this research comprises all STEM teachers and headteachers from eleven senior high schools located in the North, South, and Central Tongu districts of the Volta Region, Ghana. These districts share common characteristics, such as being primarily rural, and offer a unique context for studying the integration of digital instruction. Given that the teachers in these districts are directly involved in teaching STEM subjects, which increasingly rely on digital technologies, the insights from both teachers and headteachers are crucial to understanding the role of digital readiness, professional development, and professional learning communities in the integration of digital tools in classrooms. The rural nature of these areas, often associated with limited access to digital resources, makes the findings particularly valuable for improving digital education in similar settings.

Due to practical constraints, such as time and financial limitations, studying the entire population is challenging. Therefore, a sample, which is a subset of the population, is selected for analysis. Sampling enables researchers to conclude the population efficiently and cost-effectively, without needing to examine every individual. A well-chosen sample ensures accurate and reliable results, making it a critical element in research.

The process of selecting a sample is known as sampling, which involves choosing a smaller group from a larger population to make inferences about the entire population. Sampling techniques can be either non-random (convenient sampling) or random (probability sampling). Purposive sampling, a non-random method, was employed in this study to select participants based on their relevance and expertise. Specifically, the headteachers of the eleven schools who also serve as PLC coordinators were selected due to their leadership roles in improving digital education. One headteacher from each school was chosen to report on the school’s digital preparedness. For teacher participants, STEM teachers with at least five years of experience in the same school were selected, ensuring the participants could provide valuable insights. Teachers were grouped by the STEM subjects they taught, and 60% of teachers from each group were purposively selected.

Though purposive sampling does not provide the same generalizability as random sampling, it allows for the selection of information-rich cases that can offer deep insights into the research phenomenon. The study also incorporated probability sampling for teacher selection. Using stratified sampling, the population was divided into subgroups based on the STEM subjects, and 60% of teachers from each group were randomly selected. This method ensured a proportional representation of each STEM subject while maintaining randomness to reduce selection bias.

The combination of purposive and probability sampling techniques enabled the study to balance the need for detailed, context-specific insights with the desire for a sample that accurately represents the broader population. This approach enhances the generalizability and reliability of the research findings, making it a valuable tool for understanding the integration of digital tools in STEM education in rural Ghana.

## Results

### Background characteristics of teachers

This study provides an overview of the demographic background of the STEM teachers and headteachers involved as shown in Table 1 below. The sample included 11 headmasters and 188 STEM teachers, with 56 female participants (30%) and 132 male participants (70%). This indicates a notable gender imbalance, with males forming the majority of STEM educators in the studied districts. Most of the teachers, over 85.6% (161), are permanently employed by the Ghana Education Service, while the rest are on contract. This high level of permanent employment suggests job stability, which may support long-term engagement with professional development and the integration of digital teaching tools.

The average age of the teachers is 36.3 years, providing a perspective on the cohort’s experience and potential adaptability to new teaching methodologies. Regarding educational qualifications, the majority of the teachers, 75% (141), hold a Bachelor’s degree, while 25% (47) possess a Master’s degree or higher. This reflects a highly educated workforce, with many teachers having advanced qualifications. Such academic achievements suggest that these educators are well-prepared to engage with digital professional development and incorporate innovative digital tools into their teaching practices.

In summary, the demographic analysis highlights a predominance of male teachers, job stability among most participants, and a highly educated teaching workforce. These factors are likely to positively influence the teachers’ participation in digital professional development programs and their ability to integrate digital tools into their instruction. These insights provide a clear foundation for the subsequent analysis, helping to understand the dynamics of digital instructional integration in the schools studied.

**Table 1:** Characteristics of 188 STEM teachers.

Variables	N	%	Mean
Age	188	–	36.3
Gender			
Female	56	30	
Male	132	70	
Employment status			
Permanent	161	85.6	
Contract	27	14.36	
Educational Level			
Bachelors	141	75	
Masters or above	47	25	

### School digital preparedness

The study identifies school digital preparedness as the main independent variable, conceptualized as a latent construct. This construct is measured using a questionnaire completed by headmasters, assessing various aspects of digital tools and resources in schools. These include the availability of devices such as computers, tablets, interactive whiteboards, data projectors, internet connectivity, and technical support for teachers. The validity and reliability of the construct are confirmed through statistical analysis. Standardized factor loadings for the items range from 0.501 to 0.993, indicating a strong association between the items and the overall concept of school digital preparedness. Higher factor loadings reflect a robust relationship, confirming the reliability of the construct. Variations in mean scores and standard deviations provide insights into differences in resource availability and usage across schools, highlighting disparities in digital readiness. The measurement model demonstrates excellent construct validity, supported by a Kaiser-Meyer-Olkin (KMO) value of 0.858 and a Cronbach’s alpha of 0.878. These metrics confirm that the items effectively represent the construct, ensuring accurate measurement of school digital preparedness and its dimensions.

This analysis is presented in Table 2 below.

**Table 2:** Latent measurement characteristics for School Digital Preparedness.

Items	Mean	SD	Factor Loading
At your school, what is the total number of students in your school?	2000	N/A	0.653
Approximately, how many desktop or laptop computers are available for these students for educational purposes?	40	N/A	0.501
Approximately, how many of these desktop or laptop computers are connected to the Internet?	40	N/A	0.993
Approximately, how many tablet devices (e.g., < iPad® > , < Galaxy Book® > , < Fire®>) or e-book readers (i.e., portable devices for reading books on screen, e.g., < Amazon® KindleTM > , < Kobo>) are available for these students for educational purposes?	16	N/A	0.879
Approximately, how many interactive whiteboards are available in the school altogether?	2	N/A	0.993
Approximately, how many data projectors are available in the school altogether?	10	N/A	0.993
Approximately, how many desktop or laptop computers with internet connection are available for teachers in your school?	100	N/A	0.991
Teachers have the necessary technical and pedagogical skills to integrate <digital devices> in instruction.	2.97	0.51	0.827
Teachers have access to desktop or laptop computers for teaching or lesson planning	3.00	0.57	0.718
The school provides tablet devices (e.g., < iPad® > , < Galaxy Book® > , < Fire®>) for teaching and learning	2.78	0.73	0.864
An effective online learning support platform is available.	2.68	0.86	0.743
Teachers are provided with incentives to integrate <digital devices> in their teaching.	2.64	0.67	0.864
The school has sufficient qualified technical assistant staff.	2.99	0.62	0.719
Teachers have the necessary technical and pedagogical skills to integrate <digital devices> in instruction.	2.97	0.51	0.827

### Digital professional development

Digital professional development is one of the mediating variables in the present study and it is measured as a latent construct composed of seven items. These items capture various aspects of teachers’ participation in activities related to professional development and training in digital instructional integration. Teachers were asked about their involvement in listening to or watching recorded seminars or online courses, attending workshops on utilizing digital AI for teaching, participating in courses on using digital resources for solving real-world problems, and engaging in professional development programs that focus on evaluating the credibility of digital information. Additionally, they were asked whether they took part in in-service training courses or workshops on the use of digital resources for teaching and whether they learnt new pedagogical approaches incorporating digital resources.

The results demonstrate the construct validity of this scale. A Kaiser-Meyer-Olkin measure of sampling adequacy yielded a value of 0.779, indicating a good fit for factor analysis. The Bartlett test of sphericity was significant (χ² = 603.421, p < 0.001), confirming that the variables are intercorrelated. Furthermore, the scale’s reliability is supported by a Cronbach’s alpha of 0.7116, indicating acceptable internal consistency. The factor loadings for the seven items ranged from 0.723 to 0.852, further confirming the empirical validity of the latent construct. These results suggest that the measured items adequately capture the concept of digital professional development and its various dimensions within the context of teachers’ participation in relevant digital training activities. This analysis is presented in Table 3 below.

**Table 3:** Latent measurement characteristics for Digital Professional Development.

Items	Mean	SD	Factor Loading
Listening to or watching recorded seminars or online courses (e.g., < MOOCs>) about the use of <digital resources> for teaching	1.68	0.47	0.775
Workshops on how to utilize digital AI for teaching	1.72	0.45	0.852
Courses, workshops or in-service training on using <digital resources> to solve real-world problems (e.g., measuring the height of a building, finding directions on a map)	1.71	0.46	0.851
Professional development programs on evaluating the credibility of digital information	1.61	0.49	0.731
Course, workshop, or conference about the use of <digital resources> for teaching	2.30	0.79	0.791
In-service training courses about the use of <digital resources> for teaching	1.68	0.74	0.749
Learning new pedagogical or instructional approaches with <digital resources>	2.46	0.89	0.723

Note: Digital professional development’s Cronbach’s alpha is 0.7116, and the Kaiser-Meyer-Olkin (KMO) sampling adequacy test result is 0.779, Bartlett’s test-of-sphericity statistic χ² = 603.421 (df = 21, *p* = 0.001).

### Professional learning communities

Professional learning communities are the second mediating variable, and it is conceptualized as a latent construct in this study. The variable focuses on how teachers collaborate and engage in discussions around educational practices, particularly those related to digital instructional integration. Teachers were asked several questions to assess their participation in these communities, such as exchanging teaching materials with colleagues, discussing emerging educational technologies, and engaging in conversations regarding the learning development of specific students. The questions also covered teachers’ collaboration to ensure common standards for assessing student progress, attendance at team conferences, and using digital resources to share ideas or resources with their peers.

The results demonstrate the construct validity of this scale. A Kaiser-Meyer-Olkin (KMO) measure of sampling adequacy yielded a value of 0.6476, indicating that the data is appropriate for factor analysis. The Bartlett test of sphericity was significant (χ² = 703.36, p < 0.001), confirming that the variables are intercorrelated. The scale’s reliability is supported by a Cronbach’s alpha of 0.7423, indicating acceptable internal consistency among the items. The factor loadings for the six items ranged from 0.700 to 0.903 as presented in Table 4, further confirming the empirical validity of the latent construct. These results suggest that the measured items adequately capture the various dimensions of professional learning communities, reflecting teachers’ participation in collaborative educational activities.

**Table 4:** Latent measurement characteristics for Professional learning communities.

Items	Mean	SD	Factor Loading
Exchange teaching materials with colleagues	4.13	1.42	0.725
Discuss with colleagues about emerging educational technologies	4.70	1.43	0.835
Engage in discussions about the learning development of specific students	4.20	1.53	0.825
Work with other teachers to ensure common standards in evaluations for student progress	2.64	1.42	0.733
Use digital resources to share ideas with colleagues	3.08	1.06	0.801
Take part in professional communities of practice online	2.20	1.19	0.903

Note: The scale’s Cronbach’s alpha is 0.7423. Kaiser-Meyer-Olkin (KMO) sampling adequacy test result is 0.6476, and Bartlett’s test of sphericity statistic is χ² = 703.36 (df = 72, *p* = 0.001).

### Digital instructional integration

The dependent variable for the present study is digital instructional integration, which is conceptualized as a latent construct measured using a nineteen-item scale. Firstly, teachers responded to fourteen items on whether they use various digital technologies in their instruction, such as tutorial software, presentation software, computer-based information resources, or data logging and monitoring tools. Teachers gave their responses on a four-point Likert scale: (1) Never; (2) In some lessons; (3) In most lessons; (4) In every or almost every lesson. Secondly, teachers were asked to report on five items on the degree of emphasis they place on teaching some digital competencies, such as evaluating the credibility of digital information, sharing digital information with others, or using digital tools to work collaboratively. Teachers gave their responses on a four-point Likert scale: (1) No emphasis, (2) Little emphasis, (3) Some emphasis, (4) A lot of emphasis.

The study reports construct validity indices and standardized factor loadings of digital instructional integration, demonstrating good construct validity. Cronbach’s alpha is 0.903, and the Kaiser-Meyer-Olkin (KMO) sampling adequacy test result is 0.916. The standardized factor loadings range from 0.628 to 0.803, further suggesting the latent construct is empirically valid as presented in Table 5.

**Table 5:** Latent measurement characteristics for Digital instructional integration.

Question	Mean	SD	Factor Loading
Use digital resources to design tasks	2.299	0.786	0.6280
Use digital resources to explore new teaching methods	1.676	0.742	0.055
Use digital resources to enable student collaboration	2.461	0.893	0.6866
Use digital resources to provide feedback to students	2.171	0.856	0.7226
Use digital resources to provide access to instructional material for students who cannot physically attend class	1.736	0.892	0.5135, 0.5349
Use digital resources to communicate with parents or guardians	1.361	0.678	0.7210
Use online tools or computer-based testing to assess students’ learning	1.665	0.828	0.6399
Tutorial software or practice programs	1.709	0.813	0.7279
Digital learning games	1.519	0.728	0.7084
Word processors or presentation software (e.g., Microsoft Word, Microsoft PowerPoint)	2.281	0.826	0.7510
Spreadsheets (e.g., Microsoft Excel)	2.447	0.814	0.7990
iPads, phones	2.218	0.855	0.7124
Concept mapping software (e.g., Inspiration, Webspiration)	2.014	0.839	0.707
Data logging and monitoring tools	1.763	0.864	0.6289
Simulations and modelling software	3.203	0.798	0.7909
Social media (e.g., Facebook, Twitter)	3.348	0.790	0.7323
Communication software (e.g., email, blogs)	2.958	1.008	0.6700

Note: The scale’s Cronbach’s alpha is 0.903. Kaiser-Meyer-Olkin (KMO) sampling adequacy test result is 0.916, and Bartlett’s test of sphericity statistic is χ² = 3449.060 (df = 171, *p* = 0.001). (df = 21, *p* = 0.001).

### Partial least squares structural equation modelling (PLS-SEM)

#### Model fit index.

The researcher presents the goodness-of-fit statistics for the structural equation model, providing a comprehensive overview of how well the model fits the observed data. Key fit indices reported include the Comparative Fit Index (CFI), Tucker-Lewis Index (TLI), Root Mean Square Error of Approximation (RMSEA), and Standardized Root Mean Square Residual (SRMR). Specifically, the chi-square statistic is $\chi_{413}^{2}=\;4166.110$ (*p* < 0.001), indicating that the model differs from the saturated model. The other fit indices show CFI = 0.903, TLI = 0.984, RMSEA = 0.063, and SRMR = 0.071.

In general, CFI and TLI values of 0.9 or above are considered to demonstrate a good fit. Since both the CFI and TLI meet or exceed this threshold, it can be concluded that the model provides a reasonably good fit. Additionally, the RMSEA and SRMR have widely accepted cut off values of 0.06 and 0.10, respectively. Given that the RMSEA is 0.063 (very close to the 0.06 threshold) and the SRMR is 0.071 (a bit close to 0.10), these indices also support the conclusion that the model fits the data well. Overall, the combination of these goodness-of-fit measures suggests that the model performs satisfactorily and can be considered a good representation of the underlying relationships in the data.

### Analysis of direct effects

To test the study’s hypothesis, five pairs of direct effects were evaluated, as shown in Table 6. The Partial Least Squares Structural Equation Modelling (PLS-SEM) analysis revealed a negative and statistically significant relationship between school digital preparedness and digital integration (β = −0.10, p < 0.001). This result contradicts Hypothesis 1, which proposed a positive association between school digital preparedness and classroom digital integration.

**Table 6:** Structural equation model results for mediation analysis.

Independent Variable	Dependent Variable	Std. coefficient	Z	*p*	[95% Conf. Interval]
Direct effects					
SDP	DPD	−0.108*	−18.13	< 0.000	[-0.201, 0.096]
SDP	PLC	0.064*	10.83	< 0.000	[0.053, 0.076]
SDP	DII	0.123*	23.82	< 0.000	[0.113, 0.133]
DPD	DII	−0.420*	−83.54	< 0.000	[-0.430 -0.410]
PLC	DII	0.242*	46.02	< 0.000	[0.233, 0.253]
Indirect effect (SDP → DPD → DII)					
SDP	DII	0.045*	17.782	< 0.000	[0.040, 0.050]
Indirect effect (SDP → PLC → DII)					
SDP	DII	0.016*	10.592	< 0.000	[0.013, 0.018]
Total effect					
SDP	DII	0.184*	31.34	< 0.000	[0.071, 0.076

Model fit indices: $\chi_{413}^{2}=\;4166.110$ (*p* < 0.001), CFI = 0.903 TLI = 0.984, RMSEA = 0.063, and SRMR = 0.071. SDP: school digital preparedness, PLC: professional learning community, DPD: digital professional development, and DII: digital integration *P < 0.05.

The findings suggest that schools with higher reported levels of digital preparedness, typically defined by access to infrastructure, hardware, software, and connectivity, are paradoxically linked to lower levels of digital integration by teachers. This counterintuitive result highlights a critical gap between access and application, showing that the mere presence of technological tools does not guarantee their effective pedagogical use.

From a theoretical standpoint, this finding aligns with the Technology Acceptance Model (TAM), which emphasizes that technology adoption is shaped not only by availability but also by perceived usefulness and ease of use [95]. When teachers perceive digital tools as complex or misaligned with instructional goals, they are unlikely to integrate them, regardless of availability [96]. Similarly, the Substitution-Augmentation-Modification-Redefinition (SAMR) model illustrates that pedagogical transformation through technology requires a progression toward redefinition, often facilitated by professional development and support systems that may be lacking in infrastructure-focused schools.

This negative relationship also aligns with sociotechnical perspectives, which argue that successful digital integration depends on the interaction between technology and social systems, such as teacher beliefs, leadership, and institutional culture [97]. Technological determinism, which assumes that the mere presence of technology will transform education, overlooks these critical social elements.

A plausible explanation for the current results is the disconnect between infrastructure and teacher capability. While schools may be digitally equipped, many teachers lack the training, confidence, and pedagogical strategies to use these tools effectively [98]. When digital readiness is narrowly defined as equipment availability, it omits key components such as teacher competencies and institutional support systems.

The findings underscore the need for a holistic approach to digital transformation. Infrastructure alone is insufficient. Successful digital integration also requires sustained investment in human capital, including ongoing professional development, peer mentorship, and the establishment of professional learning communities (PLCs) where teachers can collaboratively develop their digital pedagogies [99,100].

Without these complementary elements, schools may fall into a “technology-rich but pedagogy-poor” trap, where heavy infrastructure investment fails to improve teaching or learning outcomes. Therefore, educational leaders must reconceptualize digital preparedness as an ecosystem of interdependent components, ensuring alignment between technology, pedagogy, and institutional culture to achieve sustainable digital integration.

### Indirect effects

For indirect pathways, the mediation test result presented in Table 6 demonstrates that digital professional development positively and significantly mediates the relationship between school digital preparedness and digital instructional integration with a standardized coefficient of 0.045 *(p* < 05). Upon evaluating the data collectively, it could be deduced that the indirect effect of digital professional development through digital professional development is measured to be around 27% of its direct effect (0.045/ 0.169). That is, the mediated effect is about 0.4 times as large as the direct effect of school digital preparedness on digital instructional integration. The result is statistically significant and empirically confirms the hypothesis.

On the mediating role of professional learning communities between school digital preparedness and digital instructional integration, the analysis shows that professional learning communities partially mediate the relationship with a standard coefficient of 0.016 (*p* < 05). This implies that for teachers to use available digital resources for their classroom instruction, they need to collaborate with other teachers. This collaboration enhances their readiness for the implementation of digital instructional integration. When the result is analyzed collectively, it is observed that the mediation effect of professional learning communities is about 11% of the direct effect of school digital preparedness on digital instructional integration. This also means that the mediated effect is about 0.1 times as large as the direct effect of school digital preparedness on digital instructional integration. This result is statistically significant and empirically confirms the hypothesis.

#### Total effect.

**Fig 3:**
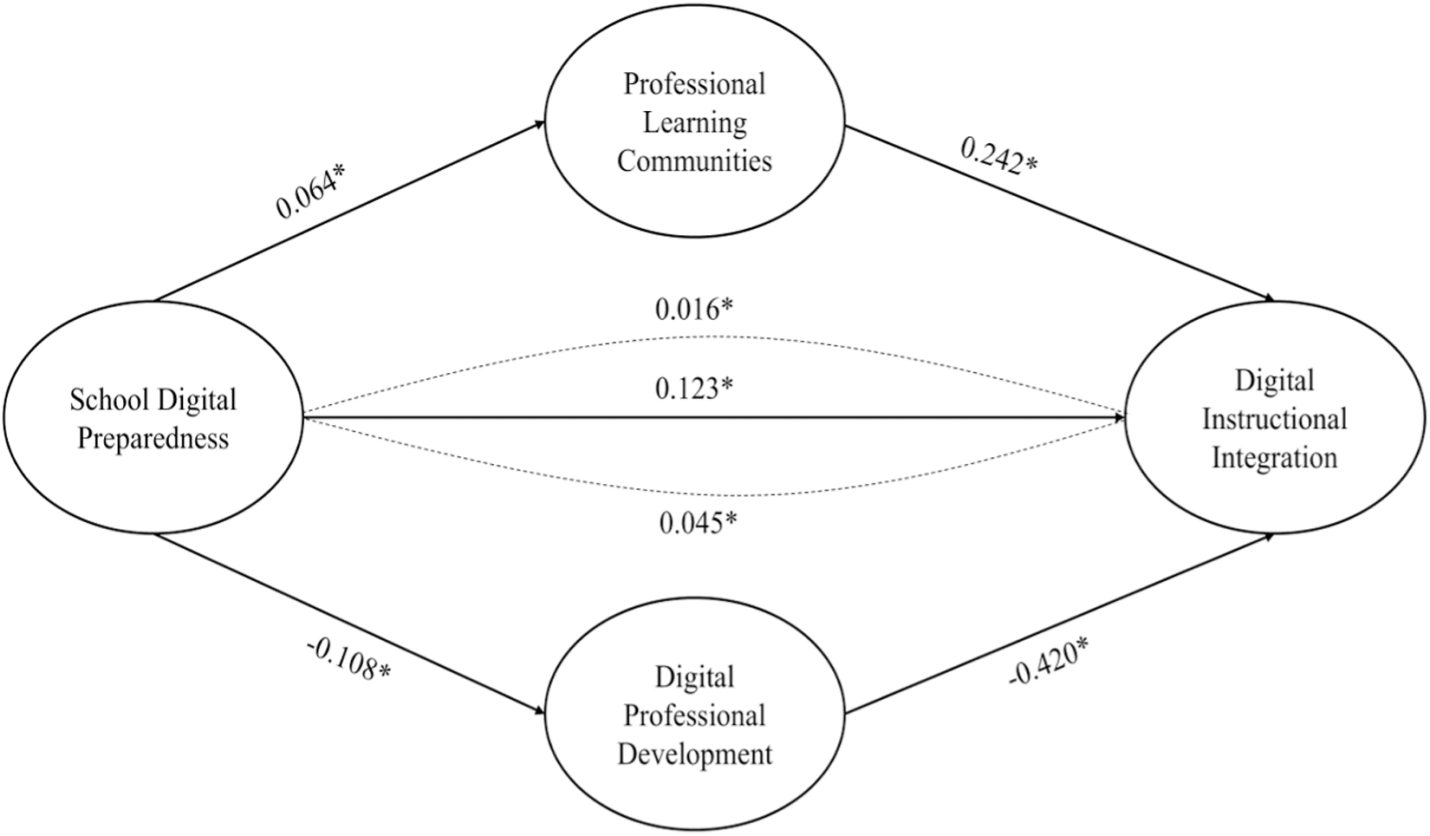
Solution Diagram of Relationships.

The total effect of school digital preparedness on digital instructional integration is a vital measure of the efficacy of school infrastructure and support in facilitating the incorporation of digital resources in education. The overall effect includes both direct and indirect effects, illustrating all the channels via which school digital preparedness impacts digital instructional integration. The total effect of school digital preparedness on digital instructional integration was determined to be 0.123 (*p* < 0.05), signifying a substantial and positive correlation. Schools exhibiting greater digital preparedness are more inclined to achieve enhanced integration of digital instructional tools within the classroom. The overall effect comprises the direct influence of school digital preparedness on digital instructional integration (0.123) and the indirect effects facilitated by professional learning communities and digital professional development. The findings emphasize that although the direct impact of school digital preparedness is significant, the contributions of professional learning communities in promoting collaboration and digital professional development in improving teachers’ digital competencies also play a crucial role in the overall effect of digital instructional integration. A visual representation of the paths and standardized solutions on the interlink of school digital preparedness, digital professional development, professional learning community, and digital instructional integration is presented in Fig 3.

## Discussions

The study provides a nuanced analysis of school digital preparedness and its role in facilitating digital instructional integration among teachers. A critical finding is the uneven availability of digital resources across schools. While some schools report high access to computers, others lag in providing essential tools such as tablets for teachers and students. This disparity raises concerns about equitable access to the technological tools necessary for interactive and personalized learning experiences [101]. Even schools with robust computer infrastructure may be limited in fostering effective digital integration if a broader range of tools is unavailable, emphasizing the need for consistent and equitable resource distribution.

The findings confirm that school digital preparedness significantly influences teachers’ acceptance and use of digital technologies, particularly among STEM teachers. Schools with robust digital infrastructure foster greater teacher confidence and willingness to adopt technology, aligning with prior research [102]. In rural areas, where personal access to digital tools is limited, schools often serve as critical providers of such resources, highlighting the importance of investment in school infrastructure to support both teachers and students [103]. Beyond resource provision, digital preparedness promotes a mindset that values innovation and aligns with contemporary teaching practices. Teachers in well-prepared schools view technology as a natural extension of their pedagogy, facilitating more widespread and effective integration of digital tools [104].

Professional Learning Communities (PLCs) also play a crucial role in enhancing digital instructional integration. Schools with high digital preparedness report increased teacher participation in PLCs, where educators exchange resources, strategies, and solutions for technology use [105,106]. These collaborative environments enable teachers to address challenges collectively, fostering continuous professional growth. PLCs not only support individual development but also encourage innovation by exposing teachers to diverse perspectives. This collaborative culture is especially valuable in rural settings, where external professional development opportunities may be limited. By promoting shared learning, PLCs significantly contribute to effective classroom technology use [107].

An unexpected finding is the negative relationship between school digital preparedness and digital professional development. Schools with high preparedness may assume teachers are already proficient, resulting in fewer training opportunities. In contrast, less-prepared schools may prioritize training to compensate for limited infrastructure. This contradiction suggests the need to align professional development initiatives with each school’s context. For well-equipped schools, training should focus on advanced applications of digital tools, while less-prepared schools should emphasize foundational training [108]. Sustained, context-specific training is essential to bridge the gap between available resources and teacher competence.

Digital professional development significantly enhances teachers’ ability to integrate technology into instruction. By equipping educators with technical skills, pedagogical strategies, and confidence, such training enables the creation of interactive and personalized learning experiences. The study underscores the need for hands-on, evolving, and targeted professional development that adapts with technological and educational advancements [109,110]. Without adequate training, even advanced tools may remain underutilized. Therefore, infrastructure investment must be paired with robust professional development to unlock technology’s full potential in education.

Both PLCs and professional development mediate the relationship between school digital preparedness and instructional integration. PLCs foster collaboration, allowing teachers to share experiences and refine strategies, while digital professional development provides the technical and pedagogical foundations for effective classroom technology use [111,112]. Together, they form a comprehensive support system for digital adoption.

The study recommends a balanced approach to digital integration. Addressing disparities in resource distribution ensures all schools have the tools for effective integration. Continuous, context-specific training empowers teachers to use these tools efficiently. Strengthening PLCs enhances integration by promoting a collaborative culture of innovation and best practice sharing. Aligning professional development with school-specific contexts ensures relevant and impactful training.

While school digital preparedness is foundational, its impact is amplified when coupled with quality professional development and PLC support. By reducing resource disparities, investing in training, and fostering collaborative communities, schools can create ecosystems that enable meaningful digital integration. These strategies enhance teacher capacity, student engagement, and learning outcomes [113,114]

### Study limitations

The present study has limitations that highlight certain constraints affecting the interpretation and generalizability of its findings. These limitations are essential to acknowledge, as they provide critical context for understanding the results and help identify directions for future research.

Firstly, the study relies on self-reported data, which may be subject to social desirability bias. Participants might have overstated their engagement with digital tools, professional learning communities, or digital instructional practices to align with perceived expectations. Although measures were taken to ensure honest responses, the potential for response bias remains and could influence the accuracy of the reported behaviours and attitudes.

Secondly, the study employs a cross-sectional design, which captures data at a single point in time. This design limits the ability to assess causality or observe trends over time. As a result, it is difficult to determine the directionality of the relationships between digital professional development, school digital preparedness, and instructional integration. A longitudinal research design would provide a more robust framework to analyse how these variables evolve and interact over time.

A significant contextual limitation lies in the geographical and demographic scope of the study. The research focuses specifically on rural senior high schools in the North, Central, and South Tongu districts of Ghana, which present a unique set of challenges, including limited infrastructure, restricted access to digital resources, and fewer professional development opportunities. These rural-specific conditions may not reflect the realities of urban or more technologically advanced schools. Therefore, while the findings provide valuable insights for similar under-resourced contexts, caution should be exercised when attempting to generalize the results to different educational environments.

Differences in infrastructure investment, teacher training systems, and cultural attitudes toward digital education in urban settings or other regions may significantly impact the applicability of these findings. Urban schools, for example, may benefit from more robust ICT infrastructure and institutional support, which could lead to different patterns of digital instructional integration. Moreover, systemic factors, such as national education policies, inequities in resource allocation, and variations in curriculum implementation, further limit the generalizability of the results beyond the studied districts.

In addition, the study focused exclusively on STEM teachers at the senior high school level. This scope excludes non-STEM teachers in basic or tertiary education, potentially missing key differences in digital integration practices across disciplines and educational levels. Including a broader range of participants in future research could help capture more diverse perspectives on digital readiness and instructional strategies.

To strengthen the generalizability and comparative value for future studies, we recommend conducting research across both rural and urban settings, with consideration for infrastructural, pedagogical, and policy differences. Longitudinal and mixed-method designs may also offer richer insights into the long-term effects of digital professional development and the evolving role of professional learning communities.

Despite these limitations, the study offers valuable, contextually grounded evidence about the factors that influence digital instructional integration in rural, resource-constrained environments. The findings serve as a foundation for informing educational policy and practice in similar contexts, while also contributing to a growing body of knowledge on digital transformation in education.

## Conclusion

The study discusses the findings, their implications for stakeholders, and the limitations of the research. The key findings highlight the significant direct effect of school digital preparedness on digital instructional integration and the mediating roles of professional learning communities and digital professional development. These findings suggest that while digital infrastructure is crucial, it is not enough to ensure effective technology integration; continuous professional development and supportive collaborative environments are also essential. Other studies may explore how these findings can inform policy decisions, resource allocation, and the development of targeted interventions to enhance digital integration in education. However, the study acknowledges limitations such as reliance on self-reported data, cross-sectional design, and geographical specificity, suggesting future research directions to address these limitations. Ultimately, the study emphasizes the need for a holistic approach to digital integration, combining digital infrastructure with continuous professional development and collaborative support through professional learning communities.

## Supporting information

S1 File Data. (ZIP)
